# Effects of agricultural practices on organic matter degradation in ditches

**DOI:** 10.1038/srep21474

**Published:** 2016-02-19

**Authors:** Ellard R. Hunting, J. Arie Vonk, C.J.M. Musters, Michiel H.S. Kraak, Martina G. Vijver

**Affiliations:** 1 Institute of Environmental Sciences (CML), Leiden University, Leiden, The Netherlands; 2 Institute for Biodiversity and Ecosystem Dynamics (IBED-AEE), University of Amsterdam, Amsterdam, The Netherlands

## Abstract

Agricultural practices can result in differences in organic matter (OM) and agricultural chemical inputs in adjacent ditches, but its indirect effects on OM composition and its inherent consequences for ecosystem functioning remain uncertain. This study determined the effect of agricultural practices (dairy farm grasslands and hyacinth bulb fields) on OM degradation by microorganisms and invertebrates with a consumption and food preference experiment in the field and in the laboratory using natural OM collected from the field. Freshly cut grass and hyacinths were also offered to control for OM composition and large- and small mesh-sizes were used to distinguish microbial decomposition and invertebrate consumption. Results show that OM decomposition by microorganisms and consumption by invertebrates was similar throughout the study area, but that OM collected from ditches adjacent grasslands and freshly cut grass and hyacinths were preferred over OM collected from ditches adjacent to a hyacinth bulb field. In the case of OM collected from ditches adjacent hyacinth bulb fields, both microbial decomposition and invertebrate consumption were strongly retarded, likely resulting from sorption and accumulation of pesticides. This outcome illustrates that differences in agricultural practices can, in addition to direct detrimental effects on aquatic organisms, indirectly alter the functioning of adjacent aquatic ecosystems.

Dead organic matter (OM) serves as a major food source sustaining diverse food webs. The consumption and decomposition of OM is an essential component of the global carbon and nutrient cycles^1^,^2^, and is governed by a complex interplay between its chemical composition, physical abrasion, microbial decomposition and consumption by invertebrates^3^,^4^,^5^,^6^. Observed positive relationships between the (functional) diversity of organisms and ecosystem functioning in a wide range of ecosystems suggest a tight link between the diversity of detritivorous microorganisms and invertebrates and the processing of OM^7^, and therefore OM degradation may serve to study and predict the quality of ecosystems in relation to anthropogenic pressures^8^,^9^,^10^.

Leaves from terrestrial plants and trees often constitute one of the major OM-inputs in aquatic ecosystems, and therefore can be important for the quantity and quality of OM available for decomposition and consumption^5^,^11^. Aquatic systems are thus prone to changes driven by land use activities at adjacent fields. This kind of land use changes are most prominent in agricultural areas. For instance, crop rotation may cause short term changes in vegetation. In addition, vegetation in agricultural areas is highly fragmented and variable over relatively short distances (cf. pastures, intensively used grasslands for dairy farms and intensively grown crops, greenhouses). Although terrestrial OM input is typically lower and autochthonous OM production (e.g. algae, macrophytes) is higher in agricultural ditches compared to woodland streams^12^, agricultural practices cause short and long term changes in terrestrial vegetation and therefore can have profound impacts on adjacent aquatic systems over various spatial scales. Changes in organic matter input due to changes in the composition and density of terrestrial vegetation have indeed been observed to affect biodiversity and ecosystem functioning in adjacent woodland streams^12^,^13^,^14^,^15^,^16^. It is thus conceivable that agricultural drainage ditches are similarly affected, yet despite the presence of a diverse detritivorous community, agricultural drainage ditches received little scientific attention^14^,^17^.

Agricultural practices inevitably involve the intensive use of pesticides, fungicides and fertilizers to increase crop yields and efficiency of production processes^18^. These chemicals are often poorly soluble and therefore quickly become associated with sediment organic particles^11^,^19^,^20^, and hence a complex mixture of organic particles, inorganic pollutants, and OM-biocide complexes is often detected^21^. These OM-complexes are subsequently subject to physical and biological processes (e.g. photolysis; microbial decomposition and macrofaunal digestion) that further complicate the chemical attributes of these OM-complexes^22^,^23^, making it difficult to predict and experimentally identify realistic effects of anthropogenic activities on ecosystem functioning^24^,^25^,^26^,^27^,^28^. Nevertheless, differences in agricultural land use practices likely have direct consequences for the subsidies of OM and chemicals to adjacent ditches, and hence it can be hypothesized that land use practices indirectly alter the functioning of the detrital food web by e.g. changing the chemical composition and nutritional value of the organic matter available to the detritivoruous community. However, a comprehensive understanding of the effect of agricultural land use on natural organic matter composition in aquatic systems and its inherent consequences for ecosystem functioning is currently lacking.

This study investigated the effect of agricultural practices on OM quality in adjacent drainage ditches by studying OM decomposition by microorganisms and consumption by invertebrates. To this end, decomposition and consumption tablets (DECOTABs) containing different natural organic matter sources related to different agricultural practices (dairy grassland farms vs. flower bulb fields) were offered to invertebrate communities within a number of highly connected agricultural drainage ditches (see Fig. 1 for a conceptual impression of the experimental setting and Fig. 2 for a map of the research area). Hyacinth bulb fields were used to represent flower bulb fields. Pesticide-free plant material of grass and hyacinth was also offered to control for the chemical composition of the OM. In addition, potential preferences for the specific OM types were tested in laboratory experiments with the keystone detritivore *Asellus aquaticus.*

## Results

Physico-chemical parameters were comparable among ditches, irrespective of agricultural land use (cf. hyacinth bulb field and grasslands; Table 1). Detritivorous richness as assessed by operational taxonomic unit (OTUs) in the agricultural ditches is presented in Fig. 3a. Ditches adjacent to grasslands and bulbfields overall contained a comparable number (~50) of unique OTUs (t-test, p > 0.4; Table 1), and on average a comparable species richness (~22, t-test, p > 0.6; Fig. 3a). The composition of OTUs did not differ between both land use types (ANOSIM, R = 0.6667, p > 0.1, Fig. 3b).

Since physico-chemicals parameters and detritivorous communities were comparable among all ditches used for the experiment, irrespective of land use practices (grass or hyacinth bulb field), consumption and decomposition measurements over the entire study area could be averaged. Consumption by invertebrates (expressed as DECOTAB mass loss over the course of the experiment) of the different OM-treatments is presented in Fig. 4. OM degradation in fine-mesh sizes often results from physical abrasion, leaching and microbial decomposition. Although we cannot rule out potential effects of physical abrasion and leaching over the entire course of the experiment, DECOTABs did not show signs of leaching in preliminary laboratory control tests. DECOTAB mass loss in the fine-mesh sized bags were therefore likely primarily controlled by microbial decomposition (cf. Fig. 4a,b). Microbial decomposition was lowest in Cellulose-only DECOTABs, and highest in DECOTABs composed of freshly cut particulate grass and hyacinth (Fig. 4a). Microbial decomposition of DECOTABs composed of POM derived from ditches adjacent to hyacinth bulb field was significantly lower compared to that of DECOTABs composed of particulate grass and hyacinth (p < 0.05) and markedly lower than, yet statistically similar to decomposition of DECOTABs composed of POM derived from ditches adjacent to grasslands. Invertebrate consumption of cellulose-based DECOTABs was significantly lower than consumption of DECOTABs composed of freshly cut particulate grass or hyacinth or POM derived from ditches adjacent to grasslands (p < 0.05, Fig. 4b). In addition, consumption of POM derived from ditches adjacent to hyacinth bulb field was significantly lower than consumption of DECOTABs composed of particulate grass or hyacinth, or POM derived from ditches adjacent to grasslands (p < 0.05), and similar to the consumption of cellulose-based DECOTABs.

OM preferences as determined by the laboratory experiment are presented in Fig. 5. At the end of the experiment, all microcosms contained comparable densities (~31 ± 5, ranging from 25–38) of *A. aquaticus* (Kruskal-Wallis, chi-squared = 13.2581, df = 9, p = 0.1513), and all treatments contained comparable populations in terms of total biomass and life stages of the individuals, ranging from juvenile to adult. Consumption of the different OM-treatments is presented in Fig. 5. Consumption of cellulose-based DECOTABs was significantly lower than that of DECOTABs composed of particulate Grass or Hyacinth, and POM derived from ditches adjacent to grasslands (p < 0.05). In addition, consumption of POM derived from ditches adjacent to hyacinth bulb field was significantly lower compared to consumption of DECOTABs composed of particulate Grass or Hyacinth, or POM derived from ditches adjacent to grasslands (p < 0.05) and comparable to the consumption of cellulose-based DECOTABs.

## Discussion

While direct effects of land use practices (e.g. the application of agricultural chemicals) are frequently studied^29^, the effect of land use practices on OM quality and inherent decomposition and consumption within detrital food webs remains poorly understood. Decomposition and consumption of specific OM types in this study were comparable throughout the study area, irrespective of agricultural practices (hyacinth bulb field vs. grass lands), but we observed a strongly retarded degradation of particulate OM derived from ditches adjacent to agricultural bulb fields. In the case of OM collected from ditches adjacent to hyacinth bulb fields, both microbial decomposition and invertebrate consumption were reduced. This was observed in the field where the entire detritivorous community had access to the DECOTABs, as well as the laboratory where DECOTABs were consumed by the key shredder *A. aquaticus*. The particulate OM used in this study was restricted to hyacinths and grass OM, and represents a complex mixture of natural OM that realistically reflects local agricultural practices, OM inputs and different stages of OM degradation, all of which were exposed to a variety of physical and biological processes (e.g. photolysis; decomposition). It thus remains speculative which mechanisms underlie the retarded decomposition and consumption of OM derived from ditches adjacent to hyacinth bulb fields, and whether the observed patterns for hyacinth bulbs are widespread among flower bulb practices. Although our assessment of physico-chemical parameters and autochthonous OM production was not exhaustive, the OM used in this study was collected from ditches that appeared to be highly comparable. Thus, except for differences related to agricultural practices, decomposition and consumption of the specific OM was consistent throughout the study area. It is thus likely that sorption and buildup of agricultural chemicals (e.g. pesticides and fungicides) adversely affected the quality and palatability of the sediment particulate OM collected from ditches adjacent to hyacinth bulb fields, rendering the OM unsuitable for both microbial decomposition and invertebrate consumption. Alternatively, it is possible that microbial decomposition was primarily retarded, and that invertebrates were indirectly affected by their frequently observed reliance on microbial conditioning of OM^4^,^30^. Irrespective of the mechanism, our study shows that, in addition to direct detrimental effects of pesticides on aquatic invertebrates, agricultural land use can thus indirectly affect ecosystem functioning by adversely affecting OM quality and therewith its decomposition by microorganisms and consumption by invertebrates.

OM composition is a major driver of microbial decomposition and invertebrate consumption, in which OM attributes such as stoichiometry and the presence of recalcitrant compounds can be very relevant in determining whether OM is a valuable nutritional resource for both microorganisms and invertebrates^31^,^32^,^33^. However, the composition of OM is often overlooked in studies aiming to resolve agricultural land use effects on the structural and functional properties of adjacent ditches. In contrast to the recalcitrant constituents (e.g. lignins, humic acids and phenolic compounds) present in natural vegetation that often comprises large numbers of trees, OM leaching from continuous tillage typically tend to show lower molecular weights, less humicity and enhanced bacterial respiration rates^34^,^35^,^36^. However, secondary metabolites in many flowering plants species are known to exhibit repelling properties, in particular toward insects^37^,^38^,^39^. While the original OM-source in this study (grass vs. hyacinths) appeared to be irrelevant for microbial OM-decomposition and invertebrate consumption in the field, invertebrate consumption in the laboratory OM preference experiment clearly indicated a that consumption of hyacinth OM was lower compared to grass OM. This difference is thus best explained by the differences in nutritional value and chemical composition of the OM, indicating that the presence of compounds exhibiting e.g. insecticidal properties could potentially contribute to a retardation of OM degradation in adjacent agricultural ditches.

The tight link between OM quality and consumption by invertebrates as observed here indicates a clear preference of invertebrates for specific substrates, in which invertebrates seem to avoid resources that are recalcitrant to degradation, low in nutritional value, or contaminated with pesticide or fungicide residues. Similar behavioral responses have been observed for detrtitivorous invertebrates in the presence of toxicants^40^,^41^,^42^. A number of invertebrate species have been observed to avoid contaminated areas and food-items^43^,^44^, which subsequently can result in reduced feeding activities and distorted organic matter processing rates^45^,^46^,^47^,^48^. The dependence of OM processing on OM quality in response to agricultural practices corroborates this notion. This study was designed to test realistic effects agricultural practices, OM inputs and different stages of OM degradation on OM degradation in agricultural drainage ditches in the presence of uniform invertebrate assemblages. The differential OM degradation observed in this study thus indicates that agricultural practices can affect the functional links between invertebrate diversity and ecosystem functioning^49^,^50^. Since the processing of OM seems consistently more sensitive than invertebrate diversity, OM-processing may serve as more sensitive, sub-lethal proxy of ecosystem integrity that reliably predicts the quality and fate of impacted aquatic ecosystems.

In conclusion, we compared OM degradation using natural OM in two distinct agricultural practices and demonstrated that agricultural practices can affect the consumption of organic matter by microorganisms and aquatic invertebrates in adjacent drainage ditches. Although we only used two types of agricultural practices, our results indicate that agricultural land-use can potentially exert strong negative effects on the functioning of ecosystems by retarding microbial and invertebrate-mediated organic matter degradation. Effects of agricultural land use variably affect OM composition and depends largely on terrestrial OM subsidies and the biological and chemical properties of both the organic matter (e.g. repelling properties of flowers) and applied agricultural chemicals (e.g. pesticides^51^). Our study illustrates that differences in agricultural practices can, in addition to the frequently observed direct detrimental effects on aquatic organisms, indirectly cascade toward altered functioning of adjacent aquatic ecosystems.

## Methods

### Description of the study area

**Figure 2:**
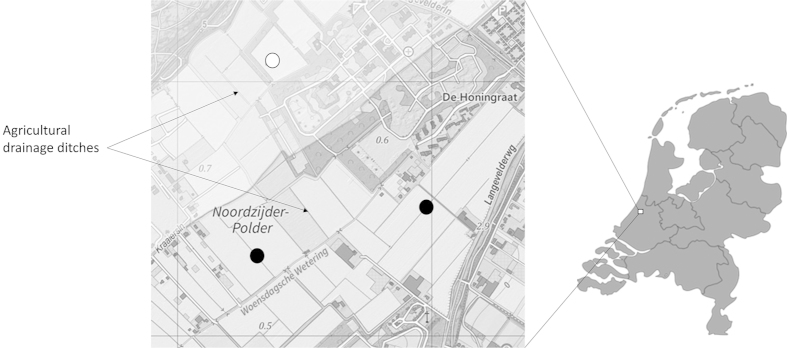
Area in the Netherlands where the OM was collected and the field experiment was performed. Inset: topographic map exemplifying the typical organization of agricultural field and adjacent drainage ditches. Ditches where natural organic matter was collected are indicated with circles. Ditches used to evaluate decomposition and consumption of the different OM-treatments included these ditches and a number of adjacent ditches. Filled circles indicate area’s with ditches surrounded by fields with flower bulb fields (total six ditches used). Open circle indicates area where ditches are surrounded by grasslands (total six ditches used). Map derived from open source geodata available from www.opentopo.nl.

**Figure 3:**
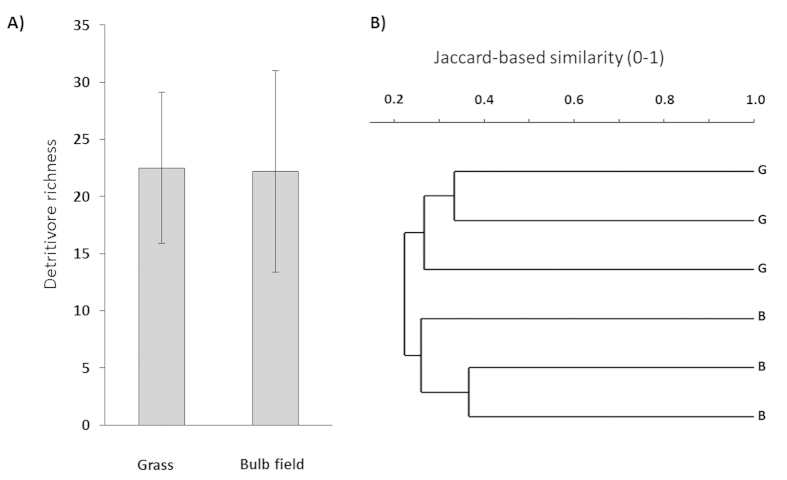
Invertebrate diversity and species composition of the ditches used to collect natural organic matter and to perform the field OM decomposition and consumption experiment. (**A**) Number of operational taxonomic units (OTU’s), expressed as detritivorous species richness, (mean ± s.d.) was not statistical different between grassland ditches and hyacinth bulb field ditches (n = 3, t-test, p > 0.4). (**B**) Invertebrate species composition, assessed as Jaccard-based similarity, was not statistical different between grassland ditches and hyacinth bulb field ditches (ANOSIM, n = 3, R = 0.6667, p = 0.1022).

**Figure 4:**
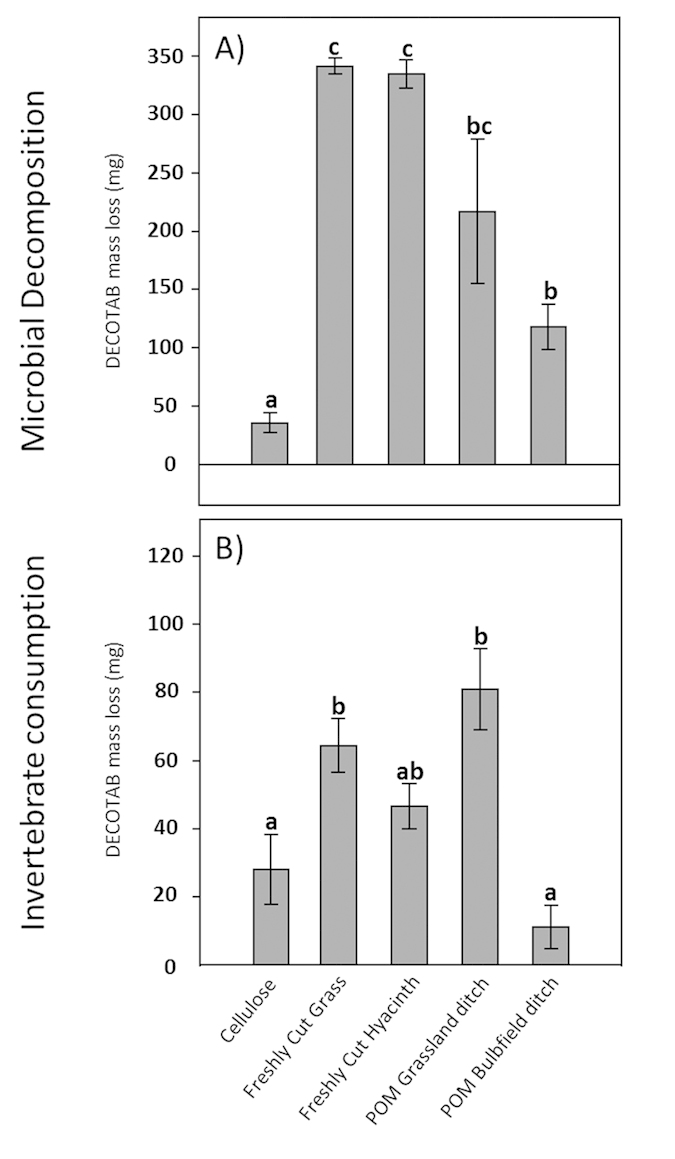
Microbial decomposition and invertebrate consumption of DECOTABs composed of different OM-sources in an OM-preference experiment in the field. (**A**) DECOTAB mass (mg) loss after 9 weeks in small mesh-sized bags representing microbial decomposition, and (**B**) DECOTAB mass (mg) (mean ± s.d.) loss after 9 weeks in large mesh-sized cages representing invertebrate consumption. DECOTAB mass loss was corrected for microbial decomposition by subtracting values obtained in (**A**). Corresponding letters indicate statistical similarity (n = 6).

**Figure 5:**
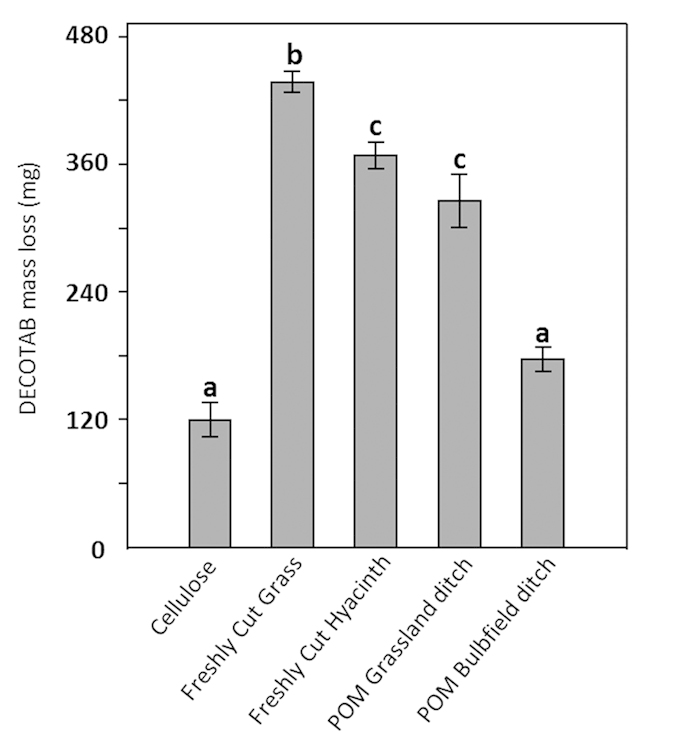
Consumption of DECOTABs composed of different OM-sources by *Asellus aquaticus* in a laboratory OM-preference experiment. Presented is total DECOTAB mass loss in (mean ± s.d.) mg after 6 weeks. Corresponding letters indicate statistical similarity (n = 9).

OM consumption was studied in an agricultural area in the south-west of the Netherlands (N52.2°; E4.1°), where agricultural drainage ditches are highly interconnected and comparable, and host a diverse and equally distributed macrofaunal community^52^ (see Fig. 2). The area is intensively used for flower bulb growing (mainly hyacinths, lilies, daffodils and tulips), but also contains several patches of grasslands used for e.g. dairy farms. Flower bulb growing involves the use and subsequent presence of various pesticides that are applied continuously in the period February-November. Flowers in the area are grown on sandy-rich soil, and drainage ditches consists mostly of medium and fine sand, facilitating leaching of high concentrations of pesticides to both groundwater and adjacent surface waters^52^. Overall, the ditches in close vicinity of agricultural fields are stagnant and only flow during pumping, and are known to have comparable physic-chemical conditions and to be moderately eutrophic^53^.

To evaluate comparability among the ditches used for the field experiment, detritivorous diversity and a number of environmental variables were determined. Temperature, pH, dissolved oxygen, and conductivity were measured with an Hach portable (2.1.0.713 HQ 40d). Phosphate, nitrate and nitrite were measured using commercially available testkits (SERA aqua Quick Test). Since macrophytes and algae have major impacts on autochthonous OM production, and habitat complexity and ditch size are major drivers of macrofaunal diversity^54^, ditches were chosen based on an equal size and equal presence and abundance of macrophytes. All chosen ditches contained patches of *Nuphar lutea* (L.), *Hydrocharis morsus-ranae* (L.), *Ceratophyllum demersum* (L.), *Spirodela polyrhiza* (L.) and Zygnematacae indet., but were not densly populated. Algal blooms do not occur during early spring in the Netherlands.

Invertebrates were sampled from the top sediment and the water column using a standard rectangular dip net. Each sampling effort was repeated three times, five meters apart from each other, within a sampling site, and all collected invertebrates were pooled. Following this sampling procedure, each ditch was sampled 3–5 times with 50 m intervals and pooled to provide an estimate of invertebrate diversity. The collected organisms were identified to the lowest possible Operational Taxonomic Unit (OTU) and evaluated for feeding habit (http://www.freshwaterecology.info/). Analysis of invertebrates were restricted to invertebrates that are known to feed on detritus. Individual-based rarefaction was used to evaluate whether sampling effort was sufficient. If rarefaction curves did not reach their asymptote, locations were excluded from further analysis. Species richness was calculated relying on OTUs, and a Jaccard-based cluster analysis and an ANOSIM with Bonferoni-corrected post hoc test was used to evaluate OTU-composition between land use types.

### Selection of OM types

Since hyacinth flower bulb fields appeared to dominate our research area during this study, they were chosen to represent this agricultural practice. Major organic matter inputs in our study area are thus grass in ditches adjacent to grasslands, and a combination of grass and hyacinths in ditches adjacent to bulb fields. To evaluate OM consumption in experiments performed in both the field and in laboratory incubations, this study was designed to simultaneously offer different replicated OM treatments: [particulate OM (POM) from ditches adjacent to grasslands], [POM from ditches adjacent to hyacinth bulb fields], [freshly collected Grass], [freshly collected pesticide free Hyacinths], and a commonly used standardized control substrate containing a primary plant constituent [Cellulose].

### POM treatments, collection and DECOTAB preparation

Since traditional approaches to study OM consumption by invertebrates (e.g. litter bags and leaf disks) carry methodological constraints to manipulate, standardize and quantify organic matter treatments in natural systems, we used the recently developed Decomposition and Consumption Tablets, DECOTABs^55^ (www.decotab.org), that embed a homogenized and standardized mixture of particulate OM, as an opportunity to overcome these constraints. DECOTABs were used to offer invertebrates the different OM treatments and approximately 100 DECOTABs were prepared for each OM-treatment following the procedures as recently described^55^. To this end, sediment particulate OM was collected from a number of ditches that were adjacent to either grasslands or hyacinth bulb fields, which were also used for the field experiment. Re-suspension and subsequent separation by differences in weight provided sediment-free particulate OM which was dried for 7 days at 45 °C. After drying, OM was sieved (500 μM–2 mm) to discard coarse material and obtain POM that was large enough to be retained within the agar matrix. DECOTABs for both land use practices (pasture POM and hyacinth POM) were prepared from 60 g/L particulate OM and 20 g/L purified agar. The agar was dissolved in dH2O and heated up to 100 °C. After cooling (<50 °C), POM was added and the solution was mixed, poured into a polycarbonate mold (diameter: 35 mm; height: 7 mm; total volume: 770 mm3), and solidified at 7 °C. Additional DECOTABs were prepared from freshly collected grass and Hyacinths obtained from an organic farmer and therefore free from agricultural chemicals. Plant material was dried for 4 days at 45 °C and ground in liquid nitrogen. DECOTABs for both grass and Hyacinth OM were prepared as described above from 60 g/L ground plant material and 20 g/L purified agar. Control cellulose DECOTABs^55^ were prepared as described above consisting of 60 g/L cellulose and 20 g/L purified agar. Prior to the experiment, a number of DECOTABs of each specific DECOTAB treatment were placed in deionized water to evaluate whether DECOTABs had a proper consistency and to visually confirm that DECOTABs were not leaching OM.

### Experimental set up of the field and laboratory experiment

For our field experiment, DECOTAB treatments were haphazardly distributed over similar ditches within the research area in April 2015. Ditches were selected in both grasslands and bulb fields growing Hyacinths to account for potentially confounding variations caused by e.g. instant effects of land use practices, invertebrate resource use histories and species compositions. A total a 12 ditches were selected and each ditch received each OM-treatment. DECOTABs were placed in cages to facilitate retrieval. The cages were constructed of circular PVC tubes (2 cm height, diameter 10 cm) and contained two different mesh sizes. The bottom mesh size was 0.5 × 0.5 cm to retain DECOTABs and facilitate access of sediment fauna (e.g. Midge larvae and Oligocheates), and the top mesh size was 2.5 × 2.5 cm to prevent consumption by e.g. fish but to allow access of larger invertebrates that can have large effects on OM degradation^56^. Four DECOTABs of a specific OM treatment were added to a single cage. Each cage received two additional inert marbles to provide extra weight necessary for placing and maintaining the cages on the bottom of the ditches. OM degradation is often primarily controlled by microorganisms under natural conditions^57^. Therefore, an additional set of four DECOTABs per specific OM treatment were enclosed in a small bag with a mesh size of 500 μm to exclude invertebrate consumption, but allow microbial decomposition, and attached to the corresponding coarse mesh cages to quantify microbial decomposition and allow correction for microbial contributions to DECOTAB mass losses. Thus, at each location, five coarse and five fine mesh-sized cages were employed, which were located approximately 5 m from each other. DECOTAB consumption was monitored visually on a weekly basis. Cages were retrieved when substantial (approx. 50%) DECOTAB mass was consumed to quantify consumption, 9 weeks after the start of the experiment. DECOTABs were subsequently rinsed, dried (70 °C for three days) and weighed. Over the course of the experiment, a number of ditches in both grasslands and bulb fields were cleared for both aquatic and bank macrophytes and experimental cages, which were therefore lost. A total of six ditches remained (three grassland ditches and three hyacinth bulb field ditches combined) as replicates for among treatment comparisons, i.e.: total DECOTAB mass loss over the course of the experiment was measured, invertebrate consumption was corrected by substracting microbial decomposition, and DECOTAB decomposition and consumption of the different OM-treatments was subsequently analyzed with a Levene’s homogeneity test, Kruskal–Wallis test, and Mann-Whitney pairwise comparisons.

An additional laboratory study was performed to evaluate whether potential differences in OM degradation were resulting from preferences for specific OM types. Microcosms consisted of rectangular (15 × 25 cm) aquaria with 3 mm inert quartz sediment and a (1:9) mixture of ditch water and deionized water, and were aerated with commercially available aquarium air pumps. To evaluate OM preference, each microcosm received one DECOTAB of each specific OM-treatment, and the microcosms were replicated 9 times. After a 2-day equilibration period, individuals of the aquatic isopod *Asselus aquaticus* were collected from nearby ditches and a total of 40 individuals ranging from juvenile to adult were added to each microcosm. These high invertebrate densities were assumed to overrule microbial contributions to DECOTAB mass loss. DECOTABs were retrieved after 6 weeks, rinsed, dried (70 °C for three days) and weighed. The number of individuals of *A. aquaticus* were counted at the end of the experiment. OM preference was subsequently determined by measuring consumption of the different OM-treatments as described above, and analyzed with a Levene’s homogeneity test, Kruskal–Wallis test and Mann-Whitney pairwise comparisons.

## Additional Information

**How to cite this article**: Hunting, E. R. *et al.* Effects of agricultural practices on organic matter degradation in ditches. *Sci. Rep.* **6**, 21474; doi: 10.1038/srep21474 (2016).

## Figures and Tables

**Figure 1:**
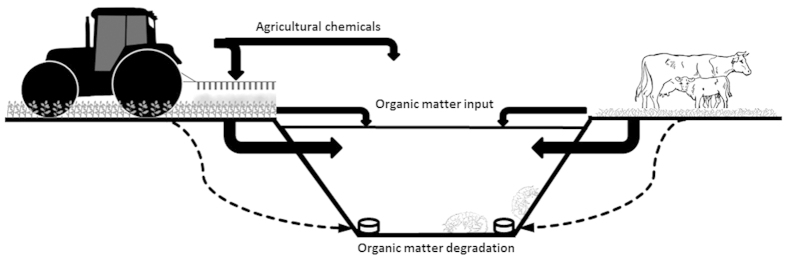
Conceptual impression of the scientific background and the experimental approach. Organic matter (OM) and agricultural chemicals from agricultural fields enter the adjacent ditches either directly or via leaching through the soil, potentially affecting the degradation of organic matter. To investigate realistic effects of agricultural practices on OM quality, decomposition and consumption tablets (DECOTABs) were prepared from natural organic material (OM) collected from agricultural drainage ditches adjacent to either hyacinth bulb field or grass lands and plant material free from agricultural chemicals to account for the chemical composition of the OM. These DECOTABs were offered to invertebrate communities in the field and a laboratory incubation of the key detritivore *Asellus aquaticus.*

**Table 1:** Physico-chemical characteristics of the ditches used to collect natural organic matter and to perform the field OM-preference experiment.

	Transparency (cm)	Conductivity (mS)	Temperature (C)	pH	Oxygen (mg/L)	NO2 (mg/L)	NO3 (mg/L)	Hardness (dGH)	CO3 (mg/L)
Grassland ditches	22.6 ± 11.5	517.9 ± 328.7	14.9 ±1.9	8.1 ± 0.3	10.4 ± 4.2	0.2 ± 0.3	2.5 ± 5.0	15.6 ± 5.8	9.8 ± 5.0
Bulbfield Ditches	33.7 ± 22.7	785 ± 44.9	15.2 ± 2.4	8.6 ± 0.3	12.4 ± 3.2	0.6 ± 1.0	5.5 ± 4.9	17.5 ± 2.6	9.5 ± 4.4

No statistical differences were observed for all measured variables (mean ± s.d.) (Student’s T-tests).
